# A Novel Calibration Method for Gyro-Accelerometer Asynchronous Time in Foot-Mounted Pedestrian Navigation System

**DOI:** 10.3390/s22010209

**Published:** 2021-12-29

**Authors:** Tianyu Chen, Gongliu Yang, Qingzhong Cai, Zeyang Wen, Wenlong Zhang

**Affiliations:** School of Instrumentation and Optoelectronic Engineering, Beihang University, Beijing 100191, China; BY2117123@buaa.edu.cn (T.C.); yanggongliu@buaa.edu.cn (G.Y.); BY1717133@buaa.edu.cn (Z.W.); SY1917107@buaa.edu.cn (W.Z.)

**Keywords:** gyro-accelerometer asynchronous time, pedestrians navigation system, calibration method, error model, zero-velocity detection

## Abstract

Pedestrian Navigation System (PNS) is one of the research focuses of indoor positioning in GNSS-denied environments based on the MEMS Inertial Measurement Unit (MIMU). However, in the foot-mounted pedestrian navigation system with MIMU or mobile phone as the main carrier, it is difficult to make the sampling time of gyros and accelerometers completely synchronous. The gyro-accelerometer asynchronous time affects the positioning of PNS. To solve this problem, a new error model of gyro-accelerometer asynchronous time is built. The effect of gyro-accelerometer asynchronous time on pedestrian navigation is analyzed. A filtering model is designed to calibrate the gyro-accelerometer asynchronous time, and a zero-velocity detection method based on the rate of attitude change is proposed. The indoor experiment shows that the gyro-accelerometer asynchronous time is estimated effectively, and the positioning accuracy of PNS is improved by the proposed method after compensating for the errors caused by gyro-accelerometer asynchronous time.

## 1. Introduction

Outdoor pedestrian positioning mainly relies on the Global Navigation Satellite System (GNSS). In indoor GNSS-denied environments, a pedestrian navigation system (PNS) is an important positioning method to locate a user’s position [1,2]. There are two kinds of technical schemes for PNS: One is the active positioning method utilizing Ultra-Wideband (UWB) [3], Bluetooth [4], ZigBee [5], Wireless Fidelity (Wi-Fi) [6], etc. The other one is the passive positioning method utilizing the Strapdown Inertial Navigation System (SINS) based on three gyros and three accelerometers [7], which makes it realizable in terms of locating the user’s position without any other external devices. A common method for the passive positioning method is to fix the MEMS Inertial Measurement Unit (MIMU) on the foot, which is also called foot-mounted pedestrian navigation system [8,9].

Limited by the low precision of MEMS inertial devices, the velocity and position of PNS will diverge rapidly over time [10]. To avoid the accumulation of velocity errors and position errors, the zero velocity updates (ZUPT) algorithm has been proposed and widely used. The velocity is approximately equal to zero when the foot touches the ground, which can be employed in a Kalman filter to correct the errors of PNS. Zero-velocity detection is one of the main factors affecting the correction effect of the ZUPT algorithm [11]. In reference [12], Barsocchi analyzed the gait characteristics and succeeded in extracting temporal gait parameters based on a sensorized footwear which contains a 3-axis gyro, a 3-axis accelerometer, a 3-axis magnetometer and five pressure sensors. References [13,14,15] present four commonly used zero-velocity detection methods which are named the stance hypothesis optimal detector, the acceleration moving variance detector, the acceleration magnitude detector, and the angular rate energy detector. The models of the four zero-velocity detection methods are compared. The experimental results showed that the detection method which utilizes the synchronous signals of gyros and accelerometers has the highest performance, and the zero-velocity detection methods perform better at slow or normal gait speed.

In order to further improve the positioning accuracy of PNS, researchers have studied the application of other sensors and constraint algorithms in PNS. In reference [16], Ruiz et al. made use of the zero angular rate updates (ZARU) algorithm assisted by MIMU and RFID with extended Kalman filter, INS algorithm, and ZUPT algorithm as the framework. The experimental results showed that the positioning error is only 1.5 m in a long-term working. Xin et al. utilized magnetometers to correct the heading errors [17]. However, it’s unpractical since the magnetic field is complex and changeable in an indoor environment. Ji et al. proposed an adaptive heading correction algorithm for suppressing magnetic interference in SINS which can correct the heading error effectively in the environments with magnetic field interference [18]. Borenstein et al. proposed a method called heuristic drift reduction (HDR), which uses the building orientation to constrain the user’s trajectory [19]. Abdulrahim et al. proposed an algorithm for generating heading measurements from basic knowledge of the orientation of the building in which the pedestrian is walking, and the results showed that the absolute position error at the final position is below 5 m, about 0.1% of the total travelled distance in the 40-min experiment with a total distance of 3 km [20]. Tanigawa et al. utilized a barometer to achieve the high-fidelity height tracking. However, in fire and smoke environments, due to the thermal noise and quantization noise, the barometer is no longer credible and useable [21]. Gu et al. proposed an integrated method utilizing gait detection and hidden Markov height estimation algorithm, to realize the effective correction of height by taking advantage of the fixed height of floors and stairs without using barometers [22]. Jing et al. proposed an adaptive collaborative positioning algorithm which selects units for the collaborative network and integrates ranging measurement to constrain inertial measurement errors. The experimental results showed that the positioning accuracy is improved by 60% compared with the traditional method [23]. Ding and Skog et al. utilized MIMU arrays to improve the positioning accuracy [24,25]. However, these above methods do not consider the effect of the asynchronization of sampling time between gyros and accelerometers.

The traditional algorithms of SINS have been analyzed under the assumption that the sampling time between gyros and accelerometers is synchronous. However, in a practical system, the gyro signals need to pass a lowpass filter, and the frequency characteristics of gyros and accelerometers are inconsistent in the common case. Particularly, most of manufacturers which produce gyros or accelerometers seldom consider the compatibility between gyros and accelerometers. Therefore, the difference in phase-frequency characteristics will lead to the asynchronization of sampling time. In addition, most systems do not synchronize the sampling time of gyros and accelerometers by using the synchronous pulse. Therefore, the incompatibility between gyros and accelerometers and the circuit defect will cause the problem of gyro-accelerometer asynchronous time. If the sampling time of gyros and accelerometers is not synchronous, the velocity errors and position errors will be accumulated continuously over time. To solve this problem, Yan et al. analyzed the effect of gyro-accelerometer asynchronous time in SINS and successfully compensated for the velocity errors after calculating the parameter of gyro-accelerometer asynchronous time [26]. Wen et al. built a model of gyro-accelerometer asynchronous time in Dual-axis RINS, and proposed a calibration method that can improve the navigation velocity accuracy and system stability under long time navigation [27]. These experimental results showed that the methods proposed by Yan and Wen succeed in calibrating the parameter of gyro-accelerometer asynchronous time and compensating for the errors in a low-dynamic environment by making IMU rotate around a single axis or double axes regularly.

Nevertheless, the existing research work of gyro-accelerometer asynchronous time is not completely applicable in PNS. Compared with SINS, the rotation of MIMU in the foot-mounted pedestrian navigation system is more complex and intense. Such a high-dynamic environment will increase the effect of gyro-accelerometer asynchronous time on pedestrian navigation. Therefore, the traditional calibration methods and error models of gyro-accelerometer asynchronous time in SINS cannot be employed in PNS. However, there are few references of gyro-accelerometer asynchronous time in PNS. In the foot-mounted pedestrian navigation system with MIMU or mobile phone as the main carrier, the gyro-accelerometer asynchronous time is likely to reach tens of milliseconds, and the gyro-accelerometer asynchronous time is changeable each time the system is turned on. Due to the gyro-accelerometer asynchronous time, the velocity error tends to be nonlinear. Therefore, the traditional error models of PNS are no longer suitable. Besides, the traditional zero-velocity detection methods which utilize gyros and accelerometers do not consider the gyro-accelerometer asynchronous time. If the sampling time of gyros and accelerometers is asynchronous, the traditional zero-velocity detection methods may lead to missing detection or false detection.

In order to solve the above problems and improve the positioning accuracy of pedestrian navigation, the gyro-accelerometer asynchronous time in PNS is studied in this paper. Compared with the known and used methodologies, this paper focuses on the calibration method of gyro-accelerometer asynchronous time in PNS which is never studied before. The proposed technology intends to provide a new way to improve the positioning accuracy of foot-mounted pedestrian navigation system in which the communication infrastructure is damaged when fire, earthquake or other emergency occurs. The main contributions in this paper can be summarized as follows: (1) A new model of gyro-accelerometer asynchronous time is built, and the effect of gyro-accelerometer asynchronous time on pedestrian navigation is analyzed. (2) A calibration method of gyro-accelerometer asynchronous time and a new zero-velocity detection method based on the rate of attitude change are proposed.

The remainder of this paper is organized as follows: Section 2 introduces the reference frame definitions. Section 3 builds an error model of gyro-accelerometer asynchronous time and analyzes the effect of gyro-accelerometer asynchronous time on pedestrian navigation. Section 4 designs a Kalman filter to calibrate the gyro-accelerometer asynchronous time and proposes a zero-velocity detection method based on the rate of attitude change. Section 5 shows the experiments and results. The discussion and conclusion are presented in Section 6.

## 2. The Reference Frame Definitions

The reference coordinate frames used in this paper are defined as follows:

$o^{i}x^{i}y^{i}z^{i}$(*i*-frame): Earth-Centered Inertially Fixed (ECIF) orthogonal reference frame.

$o^{e}x^{e}y^{e}z^{e}$(*e*-frame): Earth-Centered Earth-Fixed (ECEF) orthogonal reference frame.

$o^{b}x^{b}y^{b}z^{b}$(*b*-frame): Body orthogonal reference frame aligned with Right-Forward-Up axes of MIMU.

$o^{b^{\prime }}x^{b^{\prime }}y^{b^{\prime }}z^{b^{\prime }}$($b^{\prime }$-frame): Accelerometer orthogonal reference frame aligned with accelerometer-sensitive axes.

$o^{m}x^{m}y^{m}z^{m}$(*m*-frame): Carrier orthogonal reference frame aligned with Right-Forward-Up axes of foot.

$o^{h}x^{h}y^{h}z^{h}$(*h*-frame): Horizontal orthogonal reference frame with X-axis and Y-axis in the local horizon.

$o^{n}x^{n}y^{n}z^{n}$(*n*-frame): Navigation orthogonal reference frame aligned with local East-North-Up geodetic axes.

## 3. Gyro-Accelerometer Asynchronous Time

### 3.1. Error Model of Gyro-Accelerometer Asynchronous Time

In the foot-mounted pedestrian navigation system, the MIMU rotates with the movement of foot. In theory, the gyro outputs and accelerometer outputs are both aligned with *b*-frame, which is calculated by gyros. However, due to the gyro-accelerometer asynchronous time, the accelerometer outputs are aligned with $b^{\prime }$-frame instead of *b*-frame. Therefore, the coordinate frames aligned with gyro-sensitive axes and accelerometer-sensitive axes are no longer consistent. The angle errors between *b*-frame and $b^{\prime }$-frame can be written as:

(1) $$\left\{\begin{array}{c} \delta \theta =\theta -\theta^{\prime }\\ \delta \gamma =\gamma -\gamma^{\prime }\\ \delta \psi =\psi -\psi^{\prime } \end{array}\right.$$

where θ, γ and ψ are the pitch angle, roll angle and yaw angle from *b*-frame to *n*-frame, $\theta^{\prime }$, $\gamma^{\prime }$ and $\psi^{\prime }$ are the pitch angle, roll angle and yaw angle from $b^{\prime }$-frame to *n*-frame.

**Figure 1:**
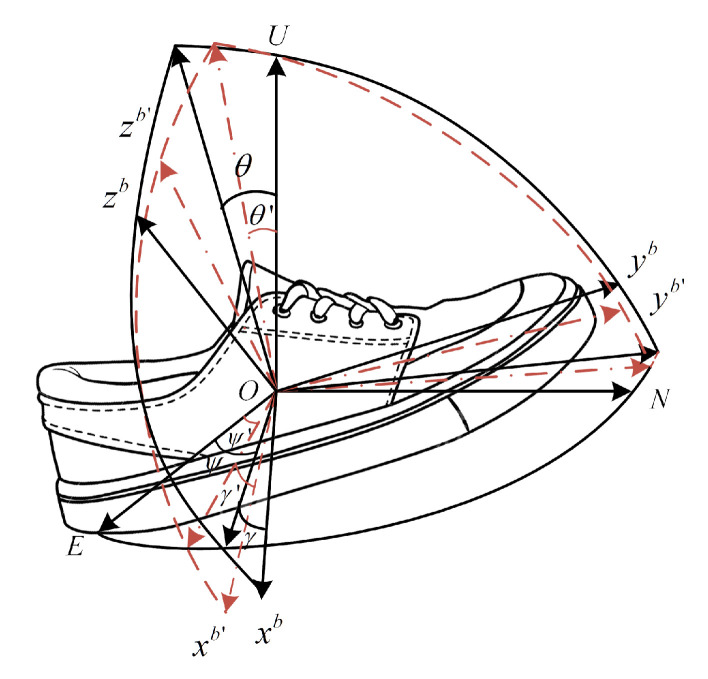
Dynamic inconsistent error of coordinate frame caused by gyro-accelerometer asynchronous time.

Figure 1 shows the dynamic inconsistent error of the coordinate frame caused by gyro-accelerometer asynchronous time. If the gyro-accelerometer asynchronous time of three axes is different, $b^{\prime }$-frame is non-orthogonal. Since the three-axis MEMS accelerometer chip is used in PNS, it can be assumed that the three gyros and three accelerometers have the same characteristics of asynchronous time. That is, the three gyros and three accelerometers have the same asynchronous time.

In this paper, $f_{}^{b^{\prime }}$ denotes the accelerometer output vector in $b^{\prime }$-frame. ${\tilde{f}}_{}^{b}$ denotes the measured specific force vector. In view of gyro-accelerometer asynchronous time, the specific force measurements are the accelerometer outputs in $b^{\prime }$-frame. Therefore, the measured specific force vector can be expressed as:

(2) $${\tilde{f}}_{}^{b}=C_{n}^{b^{\prime }}f_{}^{n}$$

where $f_{}^{n}={\left[\begin{array}{ccc} f_{E}^{n} & f_{N}^{n} & f_{U}^{n} \end{array}\right]}^{T}$ is the projection of $f_{}^{b^{\prime }}$ onto *n*-frame, and $C_{n}^{b^{\prime }}$ is the transform matrix from *n*-frame to $b^{\prime }$-frame.

The measured specific force vector in *n*-frame can be written as:

(3) $${\tilde{f}}_{}^{n}=C_{b}^{n}{\tilde{f}}_{}^{b}$$

where $C_{b}^{n}$ is the transform matrix from *b*-frame to *n*-frame.

Therefore, the specific force error vector $\delta f_{}^{n}$ caused by gyro-accelerometer asynchronous time is:

(4) $$\begin{array}{ccc} \delta f_{}^{n} & = & {\tilde{f}}_{}^{n}-f_{}^{n}\\ & \approx & C_{b}^{n}C_{n}^{b^{\prime }}f_{}^{n}-f_{}^{n}\\ & = & \left(C_{b}^{n}C_{n}^{b^{\prime }}-I\right)f_{}^{n} \end{array}$$

where

$$C_{b}^{n}=\left[\begin{array}{ccc} c_{\psi }c_{\gamma }-s_{\psi }s_{\theta }s_{\gamma } & -s_{\psi }c_{\theta } & c_{\psi }s_{\gamma }+s_{\psi }s_{\theta }c_{\gamma }\\ s_{\psi }c_{\gamma }+c_{\psi }s_{\theta }s_{\gamma } & c_{\psi }c_{\theta } & s_{\psi }s_{\gamma }-c_{\psi }s_{\theta }c_{\gamma }\\ -c_{\theta }s_{\gamma } & s_{\theta } & c_{\theta }c_{\gamma } \end{array}\right],$$

$$C_{n}^{b^{\prime }}\;=\left[\begin{array}{ccc} c_{\psi^{\prime }}c_{\gamma^{\prime }}-s_{\psi^{\prime }}s_{\theta^{\prime }}s_{\gamma^{\prime }} & s_{\psi^{\prime }}c_{\gamma^{\prime }}+c_{\psi^{\prime }}s_{\theta^{\prime }}s_{\gamma^{\prime }} & -c_{\theta^{\prime }}s_{\gamma^{\prime }}\\ -s_{\psi^{\prime }}c_{\theta^{\prime }} & c_{\psi^{\prime }}c_{\theta^{\prime }} & s_{\theta^{\prime }}\\ c_{\psi^{\prime }}s_{\gamma^{\prime }}+s_{\psi^{\prime }}s_{\theta^{\prime }}c_{\gamma^{\prime }} & s_{\psi^{\prime }}s_{\gamma^{\prime }}-c_{\psi^{\prime }}s_{\theta^{\prime }}c_{\gamma^{\prime }} & c_{\theta^{\prime }}c_{\gamma^{\prime }} \end{array}\right],$$

$$\left\{\begin{array}{c} s_{\alpha }=\sin\left(\alpha \right)\\ c_{\alpha }=\cos\left(\alpha \right) \end{array}\right.\left(\alpha =\theta ,\gamma ,\psi ,\theta^{\prime },\gamma^{\prime },\psi^{\prime }\right).$$

In order to analyze the effect of gyro-accelerometer asynchronous time on pedestrian navigation conveniently and intuitively, the movement of foot is divided into three parts, including pitch motion, roll motion, and yaw motion. The effect of pitch motion on pedestrian navigation is analyzed as follows.

The gyro-accelerometer asynchronous time is denoted as τ, and the initial three-axis attitude vector is denoted as ${\left[\begin{array}{c} 0,0,\psi_{0} \end{array}\right]}^{T}$. If the MIMU rotates around the pitch axis, Equation (2) can be rewritten as:

(5) $$\begin{array}{ccc} {\tilde{f}}_{}^{b} & = & C_{n}^{b^{\prime }}f_{}^{n}={\left(C_{\psi_{0}}C_{\theta^{\prime }}\right)}^{T}f_{}^{n}\\ \; & = & \left[\begin{array}{ccc} 1 & 0 & 0\\ 0 & c_{\theta^{\prime }} & s_{\theta^{\prime }}\\ 0 & -s_{\theta^{\prime }} & c_{\theta^{\prime }} \end{array}\right]\left[\begin{array}{ccc} c_{\psi_{0}} & s_{\psi_{0}} & 0\\ -s_{\psi_{0}} & c_{\psi_{0}} & 0\\ 0 & 0 & 1 \end{array}\right]\left[\begin{array}{c} f_{E}^{n}\\ f_{N}^{n}\\ f_{U}^{n} \end{array}\right]\\ \; & = & \left[\begin{array}{ccc} c_{\psi_{0}} & s_{\psi_{0}} & 0\\ -s_{\psi_{0}}c_{\theta^{\prime }} & c_{\psi_{0}}c_{\theta^{\prime }} & s_{\theta^{\prime }}\\ s_{\psi_{0}}s_{\theta^{\prime }} & -c_{\psi_{0}}s_{\theta^{\prime }} & c_{\theta^{\prime }} \end{array}\right]\left[\begin{array}{c} f_{E}^{n}\\ f_{N}^{n}\\ f_{U}^{n} \end{array}\right]. \end{array}$$

Substituting Equation (5) into Equation (3), ${\tilde{f}}_{}^{n}$ can be expressed as:

(6) $$\begin{array}{ccc} {\tilde{f}}_{}^{n} & = & C_{b}^{n}C_{n}^{b^{\prime }}f_{}^{n}=C_{\psi_{0}}C_{\theta }C_{n}^{b^{\prime }}f_{}^{n}\\ \; & = & \left[\begin{array}{ccc} c_{\psi_{0}} & -s_{\psi_{0}} & 0\\ s_{\psi_{0}} & c_{\psi_{0}} & 0\\ 0 & 0 & 1 \end{array}\right]\left[\begin{array}{ccc} 1 & 0 & 0\\ 0 & c_{\theta } & -s_{\theta }\\ 0 & s_{\theta } & c_{\theta } \end{array}\right]C_{n}^{b^{\prime }}f_{}^{n}\\ \; & \approx & \left[\begin{array}{ccc} 1 & 0 & \delta \theta s_{\psi_{0}}\\ 0 & 1 & -\delta \theta c_{\psi_{0}}\\ -\delta \theta s_{\psi_{0}} & \delta \theta c_{\psi_{0}} & 1 \end{array}\right]\left[\begin{array}{c} f_{E}^{n}\\ f_{N}^{n}\\ f_{U}^{n} \end{array}\right]. \end{array}$$

For the convenience of analysis, it’s assumed that people walk, go upstairs or go downstairs at normal speed. Then δθ, δγ and δψ can be considered as small angles. Therefore, δθ, δγ and δψ can be written as:

(7) $$\delta \alpha \approx \omega_{\alpha }\tau \begin{array}{cc} & ( \end{array}\alpha =\theta ,\gamma ,\psi )$$

where $\omega_{\theta }$, $\omega_{\gamma }$ and $\omega_{\psi }$ are the angular velocities along pitch axis, roll axis, and azimuth axis respectively.

Substituting Equation (7) into Equation (6), ${\tilde{f}}_{}^{n}$ can be rewritten as:

(8) $${\tilde{f}}_{}^{n}=\left[\begin{array}{ccc} 1 & 0 & \omega_{\theta }\tau s_{\psi_{0}}\\ 0 & 1 & -\omega_{\theta }\tau c_{\psi_{0}}\\ -\omega_{\theta }\tau s_{\psi_{0}} & \omega_{\theta }\tau c_{\psi_{0}} & 1 \end{array}\right]\left[\begin{array}{c} f_{E}^{n}\\ f_{N}^{n}\\ f_{U}^{n} \end{array}\right].$$

Compared with $f_{}^{n}$, the accelerometer measurement errors in *n*-frame can be expressed as:

(9) $$\left[\begin{array}{c} \delta f_{E}^{n}\\ \delta f_{N}^{n}\\ \delta f_{U}^{n} \end{array}\right]=\left[\begin{array}{c} \omega_{\theta }\tau \sin\psi_{0}f_{U}^{n}\\ -\omega_{\theta }\tau \cos\psi_{0}f_{U}^{n}\\ -\omega_{\theta }\tau \sin\psi_{0}f_{E}^{n}+\omega_{\theta }\tau \cos\psi_{0}f_{N}^{n} \end{array}\right]$$

where $\delta f_{}^{n}={\left[\delta f_{E}^{n},\delta f_{N}^{n},\delta f_{U}^{n}\right]}^{T}$ denotes the accelerometer measurement error vector in *n*-frame.

According to [28], the velocity error model is:

(10) $$\left\{\begin{array}{c} {\dot{v}}^{n}=f_{}^{n}-\left(2\omega_{ie}^{n}+\omega_{en}^{n}\right)\times v^{n}+g^{n}\\ {\dot{\tilde{v}}}^{n}={\tilde{f}}_{}^{n}-\left(2\omega_{ie}^{n}+\omega_{en}^{n}\right)\times {\tilde{v}}^{n}+g^{n} \end{array}\right.$$

where $v^{n}={\left[\begin{array}{ccc} v_{E}^{n} & v_{N}^{n} & v_{U}^{n} \end{array}\right]}^{T}$ and ${\tilde{v}}^{n}={\left[\begin{array}{ccc} {\tilde{v}}_{E}^{n} & {\tilde{v}}_{N}^{n} & {\tilde{v}}_{U}^{n} \end{array}\right]}^{T}$ are the ideal velocity vector and calculated velocity vector in *n*-frame respectively, $g^{n}$ is the local gravity vector, $\omega_{ie}^{n}$ is the angular velocity vector of earth rotation and $\omega_{en}^{n}$ is the angular velocity vector of *n*-frame relative to earth.

Therefore, the velocity error vector caused by gyro-accelerometer asynchronous time can be expressed as:

(11) $$\begin{array}{c} \delta v^{n}=\int_{0}^{t}{\dot{\tilde{v}}}_{}^{n}dt-\int_{0}^{t}{\dot{v}}_{}^{n}dt\approx \int_{0}^{t}{\tilde{f}}_{}^{n}-f_{}^{n}dt=\int_{0}^{t}\delta f_{}^{n}dt \end{array}$$

where $\delta v^{n}={\left[\begin{array}{ccc} \delta v_{E}^{n} & \delta v_{N}^{n} & \delta v_{U}^{n} \end{array}\right]}^{T}$ denotes the velocity error vector.

Substituting Equation (9) into Equation (11), the velocity error vector can be rewritten as:

(12) $$\delta v^{n}=\tau \int_{0}^{t}\omega_{\theta }\left[\begin{array}{ccc} 0 & 0 & \sin\psi_{0}\\ 0 & 0 & -\cos\psi_{0}\\ -\sin\psi_{0} & \cos\psi_{0} & 0 \end{array}\right]\left[\begin{array}{c} f_{E}^{n}\\ f_{N}^{n}\\ f_{U}^{n} \end{array}\right]dt.$$

According to Equation (12), the horizontal velocity error is:

(13) $$\begin{array}{c} \delta v_{h}^{n}=\sqrt{{\left(\delta v_{E}^{n}\right)}^{2}+{\left(\delta v_{N}^{n}\right)}^{2}}=\tau \int_{0}^{t}\omega_{\theta }f_{U}^{n}dt \end{array}$$

where the oritation is: arctan(−δvEnδvEnδvNnδvNn)=ψ0.

The vertical velocity error is:

(14) $$\begin{array}{c} \delta v_{U}^{n}=\tau \int_{0}^{t}\omega_{\theta }\left(-\sin\psi_{0}f_{E}^{n}+\cos\psi_{0}f_{N}^{n}\right)dt=\tau \int_{0}^{t}\omega_{\theta }f_{//}^{n}dt \end{array}$$

where $f_{//}^{n}$ is the equivalent velocity along the forward direction of the people’s movement. Similarly, $f_{⊥}^{n}$ denotes the equivalent velocity that is perpendicular to the forward direction of the people’s movement. $f_{⊥}^{n}$ can be expressed as:

(15) $$f_{⊥}^{n}=\cos\psi_{0}f_{E}^{n}+\sin\psi_{0}f_{N}^{n}.$$

Further, the latitude error, longitude error and height error caused by gyro-accelerometer asynchronous time can be written as follows respectively:

(16) $$\begin{array}{ccc} \delta L & = & \int_{0}^{t}\dot{\tilde{L}}dt-\int_{0}^{t}\dot{L}dt\\ & = & \int_{0}^{t}\frac{1}{R_{M}+\tilde{h}}{\tilde{v}}_{N}^{n}dt-\int_{0}^{t}\frac{1}{R_{M}+h}v_{N}^{n}dt\\ & \approx & -\frac{\tau \cos\psi_{0}}{R_{M}+h}\int_{0}^{t}\int_{0}^{t}\omega_{\theta }f_{U}^{n}dtdt, \end{array}$$

(17) $$\begin{array}{ccc} \delta \lambda & = & \int_{0}^{t}\dot{\tilde{\lambda }}dt-\int_{0}^{t}\dot{\lambda }dt\\ & = & \int_{0}^{t}\frac{\sec L}{R_{N}+\tilde{h}}{\tilde{v}}_{E}^{n}dt-\int_{0}^{t}\frac{\sec L}{R_{N}+h}v_{E}^{n}dt\\ & \approx & \frac{\tau \sin\psi_{0}\sec L}{R_{N}+h}\int_{0}^{t}\int_{0}^{t}\omega_{\theta }f_{U}^{n}dtdt, \end{array}$$

(18) $$\begin{array}{ccc} \delta h & = & \int_{0}^{t}\dot{\tilde{h}}dt-\int_{0}^{t}\dot{h}dt\\ & = & \int_{0}^{t}{\tilde{v}}_{U}^{n}dt-\int_{0}^{t}v_{U}^{n}dt\\ & = & \tau \int_{0}^{t}\int_{0}^{t}\omega_{\theta }\left(-\sin\psi_{0}f_{E}^{n}+\cos\psi_{0}f_{N}^{n}\right)dtdt \end{array}$$

where *L*, λ, and *h* are the ideal latitude, longitude and height respectively, $\tilde{L}$, $\tilde{\lambda }$, and $\tilde{h}$ are the calculated latitude, longitude and height respectively, $R_{M}$ is the radius of curvature in meridian, and $R_{N}$ is the radius of curvature in prime vertical.

### 3.2. Effects of Gyro-Accelerometer Asynchronous Time on Pedestrian Navigation

According to Equation (12), the north velocity errors can be written as follows under the assumption of $\psi =\psi_{0}$ or $\psi =\psi_{0}+\pi$:

(19) $$\left\{\begin{array}{c} {\left.\delta v_{N}^{n}\right|}_{\psi =\psi_{0}}=-\tau \cos\psi_{0}\int_{0}^{t}\omega_{\theta }f_{U}^{n}dt\\ {\left.\delta v_{N}^{n}\right|}_{\psi =\psi_{0}+\pi }=\tau \cos\psi_{0}\int_{0}^{t}\omega_{\theta }f_{U}^{n}dt. \end{array}\right.$$

Equation (19) shows that the north velocity errors are opposite in opposite directions. It’s indicated that the north velocity errors caused by gyro-accelerometer asynchronous time will cancel each other out in opposite directions. Further analysis shows that the gyro-accelerometer asynchronous time has the same effect on east velocity error.

According to Equation (13), the horizontal velocity error $\delta v_{h}^{n}$ is related to τ, $\omega_{\theta }$ and $f_{U}^{n}$. Roughly, $\omega_{\theta }$ and $f_{U}^{n}$ can be regarded as sinusoidal signals approximately with the same period. Therefore, the horizontal velocity error will be accumulated, resulting in the drift of horizontal velocity in the forward direction of people’s movement.

Equation (14) shows that the vertical velocity error is related to τ, $\omega_{\theta }$ and $f_{//}^{n}$. Since $f_{//}^{n}$ can be regarded as a sinusoidal signal approximately with the same period as $\omega_{\theta }$. Therefore, the vertical velocity error will be accumulated continuously, resulting in the rapid divergence of height eventually.

Similarly, when the MIMU rotates around the roll axis or azimuth axis, the effect of gyro-accelerometer asynchronous time on pedestrian navigation is analyzed and summarized respectively, and the results are shown in Table 1.

As shown in Table 1, affected by gyro-accelerometer asynchronous time, when the MIMU rotates around the roll axis or azimuth axis, the north velocity errors or east velocity errors are opposite in opposite directions. It means that the horizontal position errors caused by gyro-accelerometer asynchronous time will cancel each other out in opposite directions. However, when the MIMU rotates around the roll axis, the vertical velocity error will be accumulated over time, leading to the rapid divergence of height eventually. The gyro-accelerometer asynchronous time makes no difference to the vertical velocity and height if the MIMU rotates around the azimuth axis.

In addition, Table 1 also shows the relationship between velocity errors and gyro-accelerometer asynchronous time, which provides a simple and convenient way to identify the parameter of gyro-accelerometer asynchronous time.

Table 2 shows the effect of gyro-accelerometer asynchronous time on velocity errors when the MIMU rotates around the pitch axis, roll axis, or azimuth axis. In practice, the MIMU rotates around three axes driven by foot. Therefore, it’s necessary to consider the effect of pitch motion, roll motion, and yaw motion on pedestrian navigation comprehensively under different gaits. Since the MIMU mainly rotates around the pitch axis, it’s reasonable to infer that the pitch motion is the main factor affecting velocity errors. Based on the above analysis, the major characteristics of velocity errors are summarized when people walk on flat ground, go upstairs, or go downstairs. The results are shown in Table 2. The horizontal velocity errors caused by gyro-accelerometer asynchronous time will cancel each other out when people walk on a closed-loop trajectory, while the horizontal velocity error will be accumulated with time when people walk on an open-loop trajectory, resulting in decreasing the horizontal positioning accuracy. The vertical velocity and height are mainly affected by pitch motion and roll motion. Since the MIMU rotates around the pitch axis and roll axis periodically, the vertical velocity error will be accumulated over time, leading to the rapid divergence of height eventually.

### 3.3. Simulation

**Figure 2:**
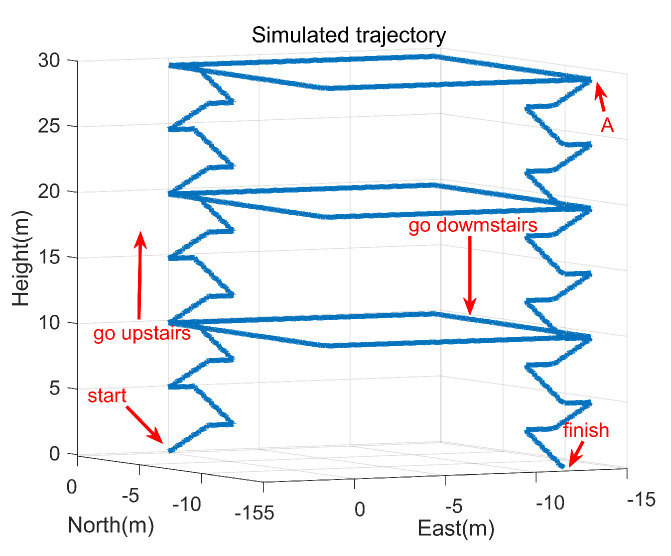
The simulated trajectory.

In order to verify the correctness of the above analyses which are shown in Table 1 and Table 2, a simulated experiment was carried out. The gyro-accelerometer asynchronous time is set to 10ms, and the designed trajectory is divided into three sorts as follows:

1. People go up to the second floor from the start, then take a walk around the hall counterclockwise. 2. People continue to go up to the third floor from the second floor, then take a walk around the hall counterclockwise. 3. people continue to go up to the fourth floor from the third floor, then take a walk around the hall counterclockwise, and go down to the first floor from point A finally. The simulated trajectory is shown in Figure 2.

The yaw motion is modeled as a rotation around azimuth axis with an uniform angular velocity. The pitch motion and roll motion are modeled as periodic sinusoidal motions in one gait cycle:

(20) $$\left\{\begin{array}{c} \theta =-\theta_{m}\sin\left(2\pi f_{step}t\right)\\ \gamma =\gamma_{m}\sin\left(2\pi f_{step}t\right) \end{array}\right.$$

where $\theta_{m}$ and $\gamma_{m}$ are the amplitudes of sinusoids, $f_{step}$ is the frequency corresponding to a gait cycle, and *t* is the working time.

**Figure 3:**
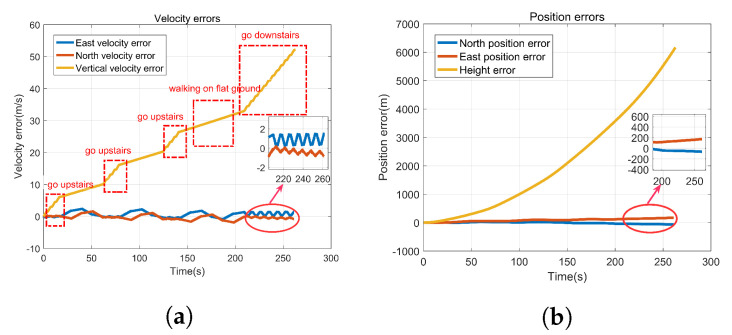
Velocity errors and position errors caused by gyro-accelerometer asynchronous time. (**a**) Velocity errors, (**b**) Position errors.

Figure 3 presents the velocity errors and position errors caused by gyro-accelerometer asynchronous time, by comparing the ideal velocity (position) with the calculated velocity (position). It shows that the velocity errors have obvious characteristics in different positions when people walk, go upstairs, or go downstairs, which meet the main characteristics described in Table 2. In addition, Figure 3b shows that the gyro-accelerometer asynchronous time has a great influence on the position, which decreases the positioning accuracy of PNS. The rapid divergence of height makes it difficult to confirm exactly which floor the person is on. Therefore, it is of great significance to calibrate the gyro-accelerometer asynchronous time and compensate for the velocity errors and position errors for improving the positioning accuracy of PNS.

## 4. A Calibration Method for Gyro-Accelerometer Asynchronous Time

Considering that the gyro-accelerometer asynchronous time is changeable, a calibration method for gyro-accelerometer asynchronous time is proposed in this chapter.

### 4.1. Error Model of Pedestrian Navigation System Based on Gyro-Accelerometer Asynchronous Time

In theory, the projection of $f_{}^{b}$ onto *n*-frame can be written as $f_{}^{n}=C_{b}^{n}f_{}^{b}$. Considering gyro-accelerometer asynchronous time, misalignment angles, and bias errors of accelerometers, the measured specific force vector in *n*-frame can be expressed as:

(21) $${\tilde{f}}_{}^{n}={\tilde{C}}_{b}^{n}{\tilde{f}}_{}^{b^{\prime }}$$

where

$${\tilde{f}}_{}^{b^{\prime }}=f_{}^{b^{\prime }}+\delta f_{}^{b},{\tilde{C}}_{b}^{n}=\left[I-\left(\phi \times \right)\right]C_{b}^{n}$$

where ${\delta f}^{b}$ is the bias error vector of accelerometers, (·×) denotes the operation of skew symmetric matrix, ϕ is the attitude error vector.

According to [27], the attitude error model is:

(22) $$\dot{\phi }=\phi \times \left(\omega_{ie}^{n}+\omega_{en}^{n}\right)+{\delta \omega }_{ie}^{n}+{\delta \omega }_{en}^{n}-C_{b}^{n}{\delta \omega }_{ib}^{b}$$

where ${\delta \omega }_{ie}^{n}$,${\delta \omega }_{en}^{n}$ and ${\delta \omega }_{ib}^{b}$ are the error vectors of $\omega_{ie}^{n}$, $\omega_{en}^{n}$ and $\omega_{ib}^{b}$ respectively, and $\omega_{ib}^{b}$ is the gyro output vector.

Ignoring the second-order small errors, Equation (21) can be rewritten as:

(23) $$\begin{array}{ccc} {\tilde{f}}_{}^{n} & = & \left[I-\left(\phi \times \right)\right]C_{b}^{n}\left(f_{}^{b^{\prime }}+\delta f_{}^{b}\right)\\ & = & \left[I-\left(\phi \times \right)\right]C_{b}^{n}f_{}^{b^{\prime }}+\left[I-\left(\phi \times \right)\right]C_{b}^{n}\delta f_{}^{b}\\ & \approx & \left[I-\left(\phi \times \right)\right]C_{b^{\prime }}^{n}C_{b}^{b^{\prime }}f_{}^{b^{\prime }}+C_{b}^{n}\delta f_{}^{b} \end{array}$$

where $C_{b}^{b^{\prime }}$ is the transform matrix from *b*-frame to $b^{\prime }$-frame. The equivalent rotation vector from *b*-frame to $b^{\prime }$-frame is donated as δϑ which can be expressed as:

(24) $$\delta \vartheta \approx \omega_{nb}^{b}\tau \approx \omega_{ib}^{b}\tau$$

where $\omega_{nb}^{b}$ is the ideal angular velocity vector.

Therefore, $C_{b}^{b^{\prime }}$ can be formed as follows by using Rodrigues rotation formula [29]:

(25) $$\begin{array}{ccc} C_{b}^{b^{\prime }} & = & M_{RV}\left(\delta \vartheta \right)\\ & = & I+\frac{\sin(\left|\delta \vartheta \right|)}{\left|\delta \vartheta \right|}\left(\delta \vartheta \times \right)+\frac{1-\cos(\left|\delta \vartheta \right|)}{{\left|\delta \vartheta \right|}^{2}}{\left(\delta \vartheta \times \right)}^{2}\\ & \approx & I+\frac{\sin(\left|{\tilde{\omega }}_{nb}^{b}\tau \right|)}{\left|{\tilde{\omega }}_{nb}^{b}\tau \right|}\left[\left({\tilde{\omega }}_{nb}^{b}\tau \right)\times \right]+\frac{1-\cos(\left|{\tilde{\omega }}_{nb}^{b}\tau \right|)}{{\left|{\tilde{\omega }}_{nb}^{b}\tau \right|}^{2}}{\left[\left({\tilde{\omega }}_{nb}^{b}\tau \right)\times \right]}^{2}\\ & \approx & I+\frac{\sin(\left|\omega_{ib}^{b}\right|\tau )}{\left|\omega_{ib}^{b}\right|}\left(\omega_{ib}^{b}\times \right)+\frac{1-\cos(\left|\omega_{ib}^{b}\right|\tau )}{{\left|\omega_{ib}^{b}\right|}^{2}}{\left(\omega_{ib}^{b}\times \right)}^{2} \end{array}$$

where $M_{RV}\left(\cdot \right)$ is the function of equivalent rotation vector.

In a practical walking process, the angular velocity is several hundred degrees per second, while the gyro-accelerometer asynchronous time is several milliseconds or tens of milliseconds in most cases. So it’s reasonable to regard δϑ as a small angle vector, and $C_{b}^{b^{\prime }}$ can be expressed as: $C_{b}^{b^{\prime }}\approx I+\left(\delta \vartheta \times \right)$. However, if people walk at high speed, such as running, sprinting, etc. The actual gyro outputs are likely to be more than one thousand degrees per second, then δϑ is no longer a small angle vector, but a large angle vector. Therefore, it’s necessary to consider the nonlinear errors caused by gyro-accelerometer asynchronous time.

According to [30], the traditional velocity error model is:

(26) $$\begin{array}{c} \delta {\dot{v}}^{n}=\left({\tilde{f}}_{}^{n}-f_{}^{n}\right)-\left(2{\tilde{\omega }}_{ie}^{n}+{\tilde{\omega }}_{en}^{n}\right)\times {\tilde{v}}^{n}+\left(2\omega_{ie}^{n}+\omega_{en}^{n}\right)\times v^{n}+\left({\tilde{g}}^{n}-g^{n}\right). \end{array}$$

Let:

(27) $$A=\frac{\sin(\left|\omega_{ib}^{b}\right|\tau )}{\left|\omega_{ib}^{b}\right|}\left(\omega_{ib}^{b}\times \right),$$

(28) $$B=\frac{1-\cos(\left|\omega_{ib}^{b}\right|\tau )}{{\left|\omega_{ib}^{b}\right|}^{2}}{\left(\omega_{ib}^{b}\times \right)}^{2}.$$

Substituting Equations (23), (25), (27) and (28) into Equation (26), the velocity error model can be rewritten as:

(29) $$\begin{array}{ccc} \delta {\dot{v}}^{n} & = & \left[I-\left(\phi \times \right)\right]C_{b^{\prime }}^{n}C_{b}^{b^{\prime }}f_{}^{b^{\prime }}+C_{b}^{n}\delta f_{}^{b}-f_{}^{n}\\ & & -\left(2{\tilde{\omega }}_{ie}^{n}+{\tilde{\omega }}_{en}^{n}\right)\times {\tilde{v}}^{n}+\left(2\omega_{ie}^{n}+\omega_{en}^{n}\right)\times v^{n}+\left({\tilde{g}}^{n}-g^{n}\right)\\ & \approx & C_{b^{\prime }}^{n}\left(I+A+B\right)f_{}^{b^{\prime }}-\left(\phi \times \right)C_{b^{\prime }}^{n}\left(I+A+B\right)f_{}^{b^{\prime }}+C_{b}^{n}\delta f_{}^{b}-f_{}^{n}\\ & & -\left(2{\tilde{\omega }}_{ie}^{n}+{\tilde{\omega }}_{en}^{n}\right)\times {\tilde{v}}^{n}+\left(2\omega_{ie}^{n}+\omega_{en}^{n}\right)\times v^{n}+\left({\tilde{g}}^{n}-g^{n}\right)\\ & \approx & \left[(C_{b}^{n}f_{}^{b^{\prime }})\times \right]\phi +C_{b}^{n}\left(A+B\right)f_{}^{b^{\prime }}+\left[C_{b}^{n}\left(A+B\right)f_{}^{b^{\prime }}\times \right]\phi \\ & & -\left(2{\delta \omega }_{ie}^{n}+{\delta \omega }_{en}^{n}\right)\times v^{n}-\left(2\omega_{ie}^{n}+\omega_{en}^{n}\right)\times \delta v^{n}+C_{b}^{n}\delta f_{}^{b}+\delta g^{n} \end{array}$$

where:

$${\tilde{\omega }}_{ie}^{n}=\omega_{ie}^{n}+{\delta \omega }_{ie}^{n},{\tilde{\omega }}_{en}^{n}=\omega_{en}^{n}+{\delta \omega }_{en}^{n},$$

$${\tilde{g}}^{n}=g^{n}+\delta g^{n},{\tilde{v}}^{n}=v^{n}+\delta v^{n}$$

where $\delta g^{n}$ is the error vector of $g^{n}$.

Equation (29) denotes the velocity error equation of PNS. The position errors can be modeled as:

(30) $$\begin{array}{ccc} \delta \dot{L} & = & \dot{\tilde{L}}-L=\frac{1}{{\tilde{R}}_{M}+\tilde{h}}{\tilde{v}}_{N}^{n}-\frac{1}{R_{M}+h}v_{N}^{n}\\ & \approx & \frac{\left(v_{N}^{n}+\delta v_{N}^{n}\right)}{\left(R_{M}+\delta R_{M}\right)+\left(h+\delta h\right)}-\frac{1}{R_{M}+h}v_{N}^{n}, \end{array}$$

(31) $$\begin{array}{ccc} \delta \dot{\lambda } & = & \dot{\tilde{\lambda }}-\dot{\lambda }=\frac{\sec\tilde{L}}{{\tilde{R}}_{N}+\tilde{h}}{\tilde{v}}_{E}^{n}-\frac{\sec L}{R_{N}+h}v_{E}^{n}\\ & \approx & \frac{\sec\left(L+\delta L\right)\left(v_{E}^{n}+\delta v_{E}^{n}\right)}{\left(R_{N}+\delta R_{N}\right)+\left(h+\delta h\right)}-\frac{\sec L}{R_{N}+h}v_{E}^{n}, \end{array}$$

(32) $$\delta \dot{h}=\dot{\tilde{h}}-\dot{h}={\tilde{v}}_{U}^{n}-v_{U}^{n}=\delta v_{U}^{n}$$

where $\delta R_{M}$ and $\delta R_{N}$ are the errors of $R_{M}$ and $R_{N}$.

Since $R_{M}>>\delta L,\delta \lambda ,\delta h$, and $R_{N}>>\delta L,\delta \lambda ,\delta h$, Equations (30) and (31) can be rewritten as:

(33) $$\delta \dot{L}\approx \frac{1}{R_{M}+h}\delta v_{N}^{n}-\frac{v_{N}^{n}}{{\left(R_{M}+h\right)}^{2}}\delta h,$$

(34) $$\delta \dot{\lambda }\approx \frac{\sec L}{R_{N}+h}\delta v_{E}^{n}+\frac{v_{E}^{n}\sec L\tan L}{R_{N}+h}\delta L-\frac{v_{E}^{n}\sec L}{{\left(R_{N}+h\right)}^{2}}\delta h.$$

### 4.2. Zero-Velocity Detection

**Figure 4:**
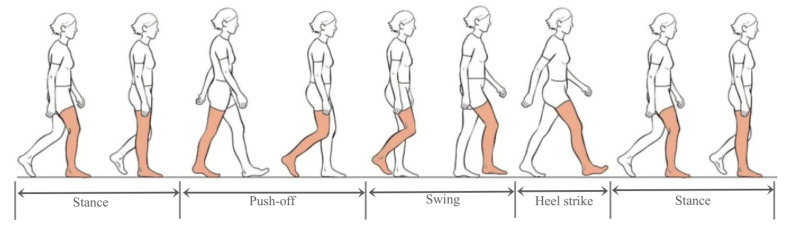
One complete gait cycle.

Figure 4 presents the details of foot movement in a complete gait cycle, including four stages, named push-off phase, swing phase, hell strike phase, and stance phase [30]. The velocity is approximately equal to zero during the sole of foot touches the ground at the stance phase.

Reference [30] presents a zero-velocity detection method named Generalized Likelihood Ratio Test (GLRT) utilizing gyros and accelerometers comprehensively. However, GLRT is utilized under the assumption that there is no gyro-accelerometer asynchronous time. If people walk, especially at high speed, although the gyros measure the angular velocities at the stance phase, the accelerometers measure the specific force before it. Thus, it will lead to missing detection or false detection if gyro-accelerometer asynchronous time is ignored. GLRT performs better when people walk at slow or normal speed. However, if people walk at high speed, the performance of GLRT seems to be not enough. Considering the effect of gyro-accelerometer asynchronous time on zero-velocity detection, this paper proposes a zero-velocity detection method based on the rate of attitude change relying on gyros only, which is applicable under various gaits.

Compared with accelerometers, the gyro sampling signals hold the most reliable information for zero-velocity detection [12]. Therefore, the detection methods utilizing gyros only perform better than the detection methods utilizing accelerometers only. As shown in Figure 4, the pitch angle and roll angle are approximately equal to zero when the sole of foot touches the ground at the stance phase. Due to the low precision of MEMS inertial devices, the attitude errors will increase gradually with time. Therefore, it’s difficult to detect the zero-velocity based on attitudes. Since the change of pitch angle or roll angle is reliable in a short term, it’s an ideal way to detect the zero-velocity by utilizing the rates of change of pitch angle and roll angle.

Considering that there are installation angle errors when the MIMU is fixed on the foot, it is necessary to compensate for the installation errors in advance. Reference [31] presents a method to calibrate the pitch installation angle error and roll installation angle error by utilizing accelerometers. A self-calibration method of yaw installation angle error is shown as follows.

The installation angle error vector is denoted as ${\left[\phi_{x},\phi_{y},\phi_{z}\right]}^{T}$. Using the chain rule of Direction Cosine Matrix (DCM) production, $C_{h}^{n}$ can be written as:

(35) $$C_{h}^{n}{=C}_{m}^{n}C_{h}^{m}.$$

Rewrite Equation (35) as:

(36) $$C_{m}^{h}={\left[{\left(C_{m}^{n}\right)}^{T}C_{h}^{n}\right]}^{T}$$

where $C_{m}^{n}$ is the transform matrix from *m*-frame to *n*-frame, $C_{h}^{n}$ is the transform matrix from *h*-frame to *n*-frame, $C_{m}^{h}$ is the transform matrix from *m*-frame to *h*-frame. Using the chain rule of DCM production, $C_{h}^{n}$ can be expressed as:

(37) $$C_{h}^{n}=C_{b}^{n}C_{h}^{b}=C_{b}^{n}C_{\phi_{x}}C_{\phi_{y}}$$

where:

$$C_{\phi_{x}}=\left[\begin{array}{ccc} 1 & 0 & 0\\ 0 & \cos(\phi_{x}) & \sin(\phi_{x})\\ 0 & -\sin(\phi_{x}) & \cos(\phi_{x}) \end{array}\right],C_{\phi_{y}}=\left[\begin{array}{ccc} \cos(\phi_{y}) & 0 & -\sin(\phi_{y})\\ 0 & 1 & 0\\ \sin(\phi_{y}) & 0 & \cos(\phi_{y}) \end{array}\right].$$

$C_{m}^{h}$ is related to $\phi_{z}$ with the following relationship:

(38) $$\tan\left(\phi_{z}\right)=\frac{{\left(C_{m}^{h}\right)}_{12}}{{\left(C_{m}^{h}\right)}_{22}}.$$

Equations (36)–(38) present the calibration method of yaw installation angle error. However, it cannot be calculated if the foot is stationary. So it’s required to make MIMU rotate to realize the calculation of $\phi_{z}$. This paper proposes a motion path of self-calibration for the yaw installation angle error, which is shown as follows.

It is assumed that the initial attitude vector is ${\left[\begin{array}{ccc} 0 & 0 & \varphi_{0} \end{array}\right]}^{T}$. When the person is walking along a straight line, the rotation angle vector from *m*-frame to *n*-frame is denoted as $\phi_{1}\approx {\left[\begin{array}{ccc} \theta_{1} & \gamma_{1} & \varphi_{1} \end{array}\right]}^{T}$ which represents the actual attitudes of the foot, and the rotation angle vector from *h*-frame to *n*-frame is denoted as $\phi_{2}\approx {\left[\begin{array}{ccc} \theta_{2} & \gamma_{2} & \varphi_{2} \end{array}\right]}^{T}$ which can be obtained from: $M_{RV}\left(\phi_{2}\right)=C_{b}^{n}C_{\phi_{x}}C_{\phi_{y}}$. Since the person is walking along a straight line, the change of yaw angle can be regarded as a small angle. It’s reasonable to make an approximation that $\theta_{1}\approx \theta_{2}$, and $\gamma_{1}\approx \gamma_{2}$. Then, $\phi_{1}$ can be expressed as:

(39) $$\phi_{1}\approx {\left[\begin{array}{ccc} \theta_{2} & \gamma_{2} & \varphi_{0} \end{array}\right]}^{T}.$$

Therefore, Equation (36) can be rewritten as:

(40) $$\begin{array}{c} C_{m}^{h}\approx {\left\{{\left[M_{RV}\left(\phi_{1}\right)\right]}^{T}C_{b}^{n}C_{\phi_{x}}C_{\phi_{y}}\right\}}^{T}. \end{array}$$

**Figure 5:**
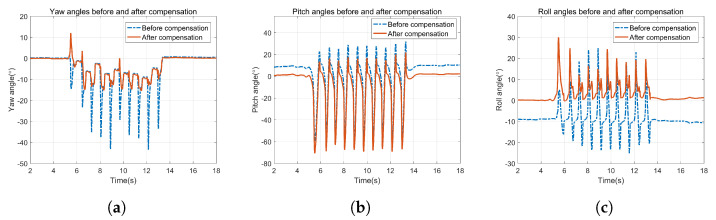
Attitudes before and after compensating for the installation angle errors. (**a**) Yaw angle, (**b**) Pitch angle, (**c**) Roll angle.

Figure 5 presents the attitudes before and after compensating for the installation angle errors. It shows that the attitudes are corrected after compensating for the installation angle errors. The proposed calibration method of installation angle errors improves the recognition and characteristic of gait successfully.

**Figure 6:**
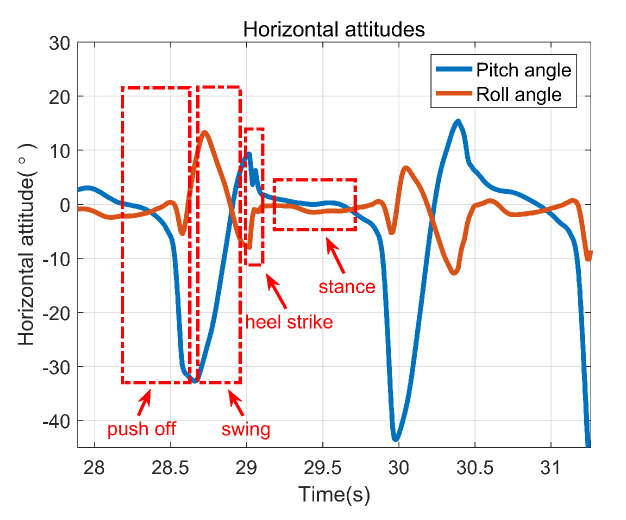
Horizontal attitudes in one gait cycle.

Figure 6 presents the horizontal attitudes in a complete gait cycle. It’s indicated that the horizontal attitudes show different characteristics in different stages significantly. This paper proposes a zero-velocity detection method based on the rate of attitude change, which is shown as follows.

A fixed length sliding window is used, and the increments of pitch angle and roll angle of MIMU in $\left[t_{k},t_{k+N}\right]$ are denoted as:

(41) $$\left\{\begin{array}{c} \Delta \theta_{i}=\theta_{i+1}-\theta_{i}\left(i=k,k+1,...,k+N\right)\\ \Delta \gamma_{i}=\gamma_{i+1}-\gamma_{i}\left(i=k,k+1,...,k+N\right) \end{array}\right.$$

where *N* is the fixed length of sliding window.

If $\Delta \theta_{i}$ and $\Delta \gamma_{i}$ are less than the thresholds, it is indicated that the zero-velocity is detected. The judgment can be expressed as follows:

(42) $$C_{1}=\left\{\begin{array}{c} 1\forall t_{i}\in \left[t_{k},t_{k+N}\right],\left|\Delta \theta_{i}\right|<G_{\theta }\\ 0others \end{array}\right.,$$

(43) $$C_{2}=\left\{\begin{array}{c} 1\forall t_{i}\in \left[t_{k},t_{k+N}\right],\left|\Delta \gamma_{i}\right|<G_{\gamma }\\ 0others \end{array}\right.$$

where $G_{\theta }$ and $G_{\gamma }$ are the thresholds.

If $C_{1}\&C_{2}=1$, the zero-velocity is detected.

**Figure 7:**
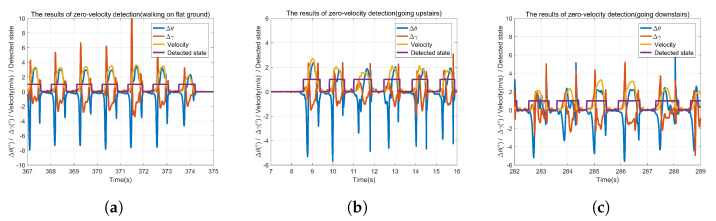
The results of zero-velocity detection. (**a**) Walking on flat ground, (**b**) Going upstairs, (**c**) Going downstairs.

Figure 7 presents the results of zero-velocity detection under different gaits. The zero value of the purple line means that the foot is stationary at that moment, while the non-zero value of the purple line means that the foot is non-stationary at that moment. It shows that the zero-velocity is detected effectively by utilizing the proposed zero-velocity detection method.

### 4.3. Kalman Filter Design

**Figure 8:**
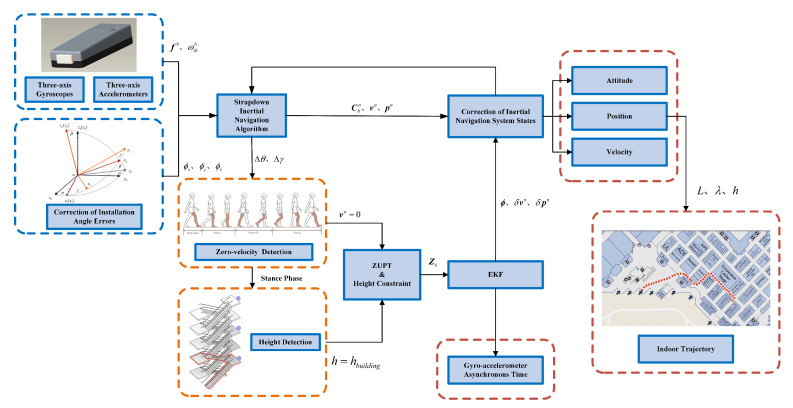
The integrated framework block diagram.

Equation (29) shows that the velocity error model is nonlinear. Therefore, an Extended Kalman Filter (EKF) is utilized to estimate the parameter of gyro-accelerometer asynchronous time in this paper. The integrated framework block diagram of PNS is shown in Figure 8.

The state vector for Kalman filter is:

(44) $$X={\left[\begin{array}{cccccc} \phi^{T} & {\left(\delta v^{n}\right)}^{T} & {\left({\delta p}^{n}\right)}^{T} & {\left(\varepsilon^{b}\right)}^{T} & {\left(\nabla^{b}\right)}^{T} & \tau \end{array}\right]}^{T}$$

where ${\left(\delta p^{n}\right)}^{T}={\left[\begin{array}{ccc} \delta L & \delta \lambda & \delta h \end{array}\right]}^{T}$ is the position error vector, ${\left(\varepsilon^{b}\right)}^{T}={\left[\begin{array}{ccc} \varepsilon_{x}^{b} & \varepsilon_{y}^{b} & \varepsilon_{z}^{b} \end{array}\right]}^{T}$ is the bias error vector of gyros, and ${\left(\nabla^{b}\right)}^{T}={\left[\begin{array}{ccc} \nabla_{x}^{b} & \nabla_{y}^{b} & \nabla_{z}^{b} \end{array}\right]}^{T}$ is the bias error vector of accelerometers. ${\left(\varepsilon^{b}\right)}^{T}$, ${\left(\nabla^{b}\right)}^{T}$ and τ have the following relationship:

(45) $$\left\{\begin{array}{c} {\dot{\varepsilon }}^{b}=0\\ {\dot{\nabla }}^{b}=0\\ \dot{\tau }=0 \end{array}\right..$$

The state transition equation can be expressed as:

(46) $$\dot{X}=f\left(X\right)+\mathrm{GW}$$

where $f\left(X\right)$ is nonlinear vector function, G is process noise coupling matrixr, W is process noise vector.

The measurement equation can be written as:

(47) Z=HX+V

where V is measurement noise vector, Z and H are measurement vector and measurement matrix respectively.

In this paper, the height estimation method proposed by Gu et al. is utilized to estimate accurate height measurements for EKF [22]. Therefore, Z and H can be expressed as:

(48) $$Z=\left[\begin{array}{c} {\tilde{v}}_{}^{n}-0\\ {\tilde{h}}_{}-h_{building} \end{array}\right],$$

(49) $$H=\left[\begin{array}{ccccc} 0_{3\times 3} & I_{3\times 3} & 0_{3\times 2} & 0_{3\times 1} & 0_{3\times 7}\\ 0_{1\times 3} & 0_{1\times 3} & 0_{3\times 2} & 1 & 0_{1\times 7} \end{array}\right]$$

where $h_{building}$ is the height measurement calculated by the height estimation method.

Discretizing Equations (46) and (47), the state transition equation and measurement equation can be rewritten as:

(50) $$\left\{\begin{array}{c} X_{k}=f\left(X_{k-1}\right)+\Gamma_{k-1}W_{k-1}\\ Z_{k}=H_{k}X_{k}+V_{k} \end{array}\right.$$

where $W_{k-1}$ and $V_{k}$ are zero-mean Gaussian white noise vector sequences, both of which are not correlated. The feedback compensation of EKF can be expressed as:

(51) $$\left\{\begin{array}{c} C_{b}^{n}=\left[I_{3\times 3}+\left(\phi \times \right)\right]{\tilde{C}}_{b}^{n}\\ v^{n}={\tilde{v}}^{n}-\delta v^{n}\\ h=\tilde{h}-\delta h \end{array}\right..$$

## 5. Experiments and Analysis

**Figure 9:**
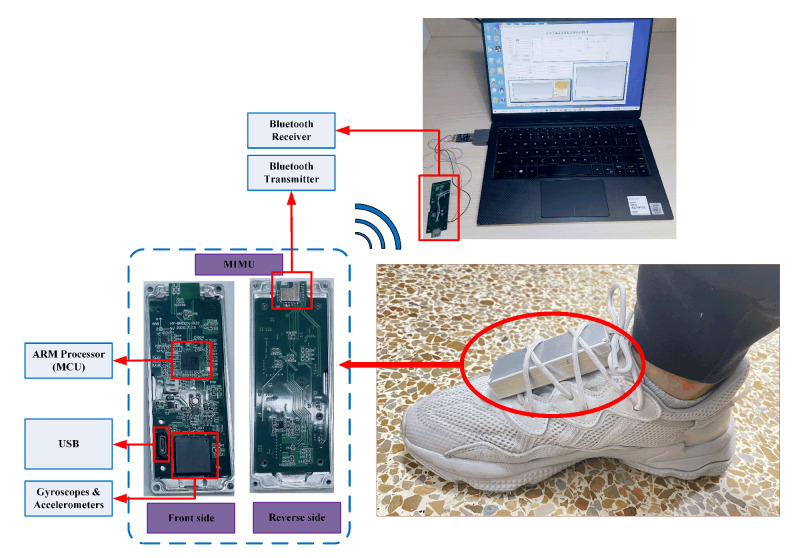
Data acquisition conditions.

The experiment was conducted in a building in Jimei District, Xiamen. As shown in Figure 9, to collect the data, the MIMU was fixed on the instep of the right foot. Then the experimenter walked on a designed trajectory that contains different gaits, including walking on flat ground, going upstairs, and going downstairs. While the experimenter was walking indoor, the MIMU sent the gyro outputs and accelerometer outputs to the Bluetooth receiver connected to a PC through the Bluetooth transmitter. In this experiment, a self-designed MIMU which contains three gyros and three accelerometers with large ranges is used. The performance of the MIMU is shown in Table 3.

**Figure 10:**
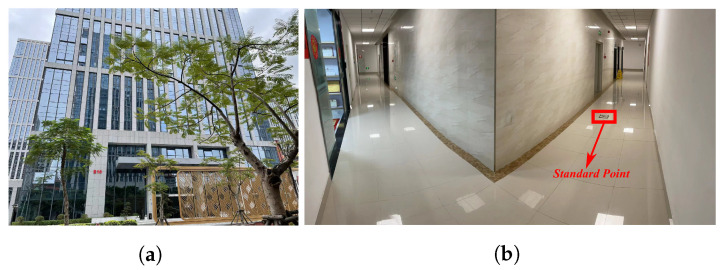
Experimental environment. (**a**) Outdoor environment, (**b**) Indoor environment.

The experimental environment is shown in Figure 10. The height of the floors is 4 m, and a start point and three standard points are set up in advance according to the engineering drawing of the building. Their relative coordinates in the building can be expressed as follows:

$P_{0}\left(0,0,0\right)$: the start point on the first floor; $P_{1}\left(0,0,8\right)$: standard point 1 on the third floor; $P_{2}\left(0,0,12\right)$: standard point 2 on the fourth floor; $P_{3}\left(0,0,8\right)$: standard point 3 on the third floor coincided with standard point 1.

The designed trajectory can be summarized as follows: The experimenter went up to the third floor from $P_{0}$, and took a walk around the hall counterclockwise to reach $P_{1}$. Then the experimenter went up to the fourth floor, and took a walk around the hall clockwise to reach $P_{2}$. Finally, the experimenter went down to the third floor, and took a walk around the hall counterclockwise to reach $P_{3}$. The total travelled distance is about 300 m.

**Figure 11:**
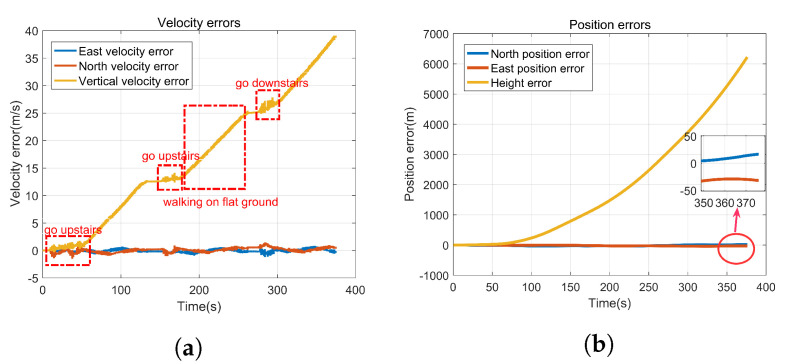
Velocity errors and position errors caused by gyro-accelerometer asynchronous time in the experiment. (**a**) Velocity errors, (**b**) Position errors.

After data collection is completed, the gyro sampling signals and accelerometer sampling signals are compared offline. The estimation of gyro-accelerometer asynchronous time is about 10 ms. A method named interpolation is utilized to compensate for the errors caused by gyro-accelerometer asynchronous time then. The error curves are drawn in Figure 11, by comparing the compensated velocity (position) with the uncompensated velocity (position). Figure 11 shows that the characteristics of velocity errors and position errors caused by gyro-accelerometer asynchronous time meet the results described in Table 1 and Table 2. It further proves the correctness of the above analyses.

Reference [32] presents the ZUPT algorithm and the height constraint algorithm, both of which show excellent performance in PNS. To evaluate the performance of the proposed calibration method, four methods are designed in this paper. Method 1 utilizes the ZUPT algorithm with gyro-accelerometer asynchronous ignored. Method 3 utilizes the ZUPT algorithm and the height constraint algorithm with gyro-accelerometer asynchronous ignored. Method 2 and method 4 are designed to verify the effectiveness and feasibility of the proposed calibration method by comparing the positioning accuracy with method 1 and method 3. The designed methods are shown in Table 4 in detail.

**Figure 12:**
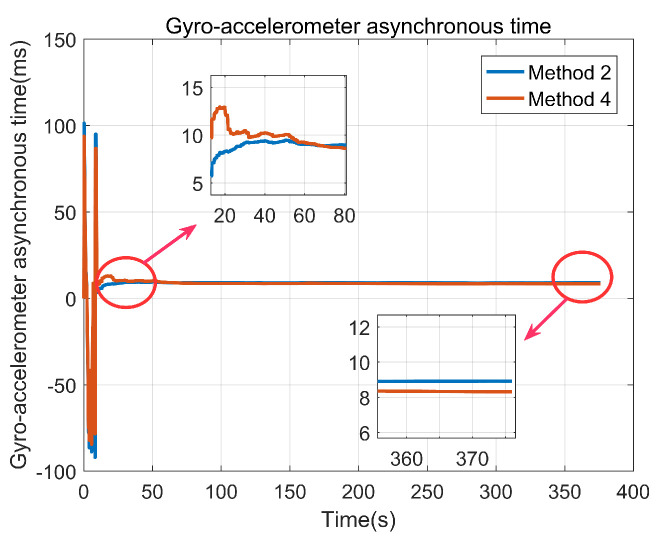
Gyro-accelerometer asynchronous time estimation.

Figure 12 presents the estimations of gyro-accelerometer asynchronous time with method 2 and method 4. Since method 1 and method 3 do not consider the gyro-accelerometer asynchronous time, the information about method 1 or method 3 is not presented in Figure 12. It shows that the estimation of gyro-accelerometer asynchronous time with method 2 is about 8.92 ms, and the estimation of gyro-accelerometer asynchronous time with method 4 is about 8.31 ms. These results are very close to the offline calculated estimation. Moreover, Figure 12 also shows that the curves are convergent after about 60 s due to the complex movement of MIMU driven by foot, indicating that the gyro-accelerometer asynchronous time can be inspired in a relatively short time.

**Figure 13:**
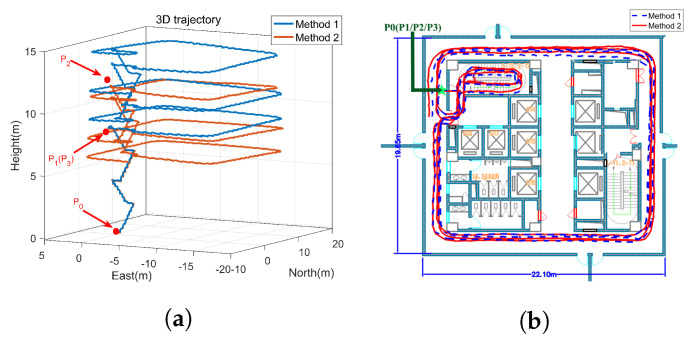
The trajectories with method 1 and method 2. (**a**) 3D trajectory, (**b**) 2D trajectory.

**Figure 14:**
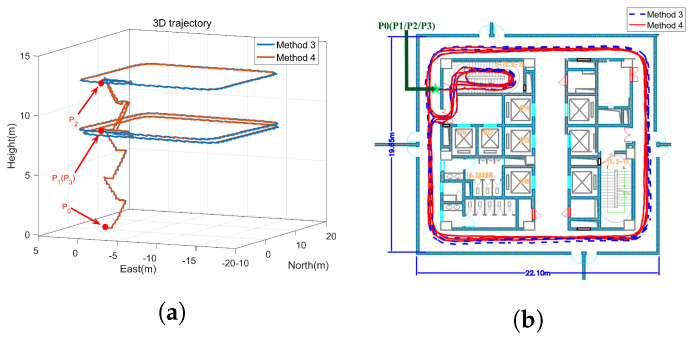
The trajectories with method 3 and method 4. (**a**) 3D trajectory, (**b**) 2D trajectory.

Figure 13 presents the calculated trajectories with method 1 and method 2, and Figure 14 presents the calculated trajectories with method 3 and method 4. Figure 13a shows that the height error with method 1 is positive while the height error with method 2 is negative. Further analysis shows that the height error with method 2 is affected by the low precision of MEMS inertial devices and the computational errors of inertial navigation algorithm, while the height error with method 1 is also affected by the gyro-accelerometer asynchronous time. Therefore, the height errors with method 1 and method 2 are opposite. Figure 13b shows that the calculated trajectory with method 2 is more consistent with the real trajectory than the calculated trajectory with method 1. In addition, as shown in Figure 14, compared with the calculated trajectories with method 1 and method 2, method 3 and method 4 have a higher positioning accuracy. The height constraint algorithm makes it possible to confirm exactly which floor the experimenter is on.

**Figure 15:**
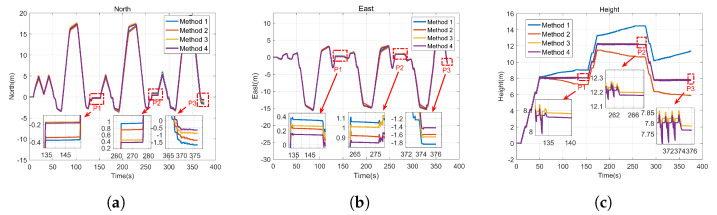
The calculated positions by different methods. (**a**) North, (**b**) East, (**c**) Height.

**Figure 16:**
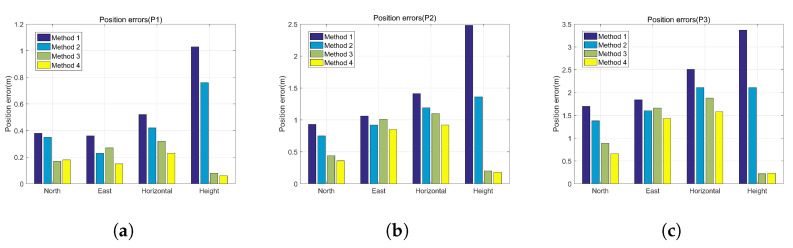
The position errors at three standard points. (**a**) Standard point 1, (**b**) Standard point 2, (**c**) Standard point 3.

In order to further verify the effectiveness of the proposed calibration method, the positioning errors are compared. Figure 15 presents the position curves by four methods respectively. Figure 16 presents the position errors by four methods at three standard points. The details of the position errors are shown in Table 5, Table 6 and Table 7. The experimental results show that the horizontal position errors with method 1 are 0.52 m, 1.41 m and 2.51 m, accounting for 0.50%, 0.70% and 0.84% of the total travelled distance respectively, and the horizontal position errors with method 2 are 0.42 m, 1.19 m and 2.11 m, accounting for 0.40%, 0.59% and 0.70% of the total travelled distance respectively. Compared with method 1, the horizontal positioning accuracy with method 2 increases by 23.81%, 18.49% and 18.96% respectively. The horizontal position errors with method 3 are 0.32 m, 1.10 m and 1.88 m, accounting for 0.31%, 0.54% and 0.64% of the total travelled distance respectively, and the horizontal position errors with method 4 are 0.23 m, 0.92 m and 1.58 m, accounting for 0.22%, 0.46% and 0.53% of the total travelled distance respectively. Compared with method 3, The horizontal positioning accuracy with method 4 increases by 39.13%, 19.57% and 18.99% respectively. Therefore, the results prove that the horizontal positioning accuracy is improved after compensating for the errors caused by gyro-accelerometer asynchronous time. Further analysis shows that the velocity errors are only corrected at the stance phase by using the ZUPT algorithm. Since the zero-velocity interval accounts for a small part of a complete gait cycle, the velocity errors and position errors will be accumulated continuously. Although the horizontal velocity errors caused by gyro-accelerometer asynchronous time will cancel each other out partly on a closed-loop trajectory, the complex movement of foot will still lead to the drift of velocity. Therefore, the horizontal positioning accuracy can be improved by compensating for the errors caused by gyro-accelerometer asynchronous time.

Besides, compared with method 1, the vertical positioning accuracy with method 2 increases by 0.27 m, 1.12 m and 1.26 m respectively at $P_{0}$, $P_{1}$ and $P_{2}$, indicating that the proposed calibration method can effectively compensate for the height error caused by gyro-accelerometer asynchronous time when using the ZUPT algorithm. However, the improvement of vertical positioning accuracy with method 4 is limited, due to the effective correction of height when using the height constraint algorithm.

## 6. Discussion and Conclusions

The experimental results show that the gyro-accelerometer asynchronous time can be estimated in a short time due to the high-dynamic environments. The proposed calibration method performs well in estimating the parameter of gyro-accelerometer asynchronous time. The positioning accuracy can be improved effectively after compensating for the errors caused by gyro-accelerometer asynchronous time. Since the position errors caused by gyro-accelerometer asynchronous time will be accumulated over time, the proposed calibration method contributes to improving the stability of PNS in a long time.

In the foot-mounted pedestrian navigation system with MIMU or mobile phone as the main carrier, the difference of phase-frequency characteristics between gyros and accelerometers will lead to the asynchronization of sampling time, resulting in the accumulation of velocity errors and position errors. To solve this problem, in this paper, an error model of gyro-accelerometer asynchronous time is built. The effect of gyro-accelerometer asynchronous time on pedestrian navigation and the main characteristics of velocity errors under different motions are analyzed. A filtering model is designed to calibrate the gyro-accelerometer asynchronous time via a Kalman filter. To avoid the missing detection and false detection, a zero-velocity detection method based on the rate of attitude change is proposed. The results of the 300 m-long experiment show that the gyro-accelerometer asynchronous time is estimated effectively and the positioning accuracy is improved after compensating for the errors caused by gyro-accelerometer asynchronous time. Furthermore, the gyro-accelerometer asynchronous time is caused by hardware, while the proposed method reduces the negative effect of gyro-accelerometer asynchronous time by software. Therefore, for the MIMUs with gyro-accelerometer asynchronous time, the proposed method makes it possible to apply them in PNS, which reduces the cost of pedestrian navigation and improves the reliability of MIMUs or mobile phones in PNS. Therefore, it can be concluded that the proposed calibration method works well in the foot-mounted pedestrian navigation system, and the study of gyro-accelerometer asynchronous time provides a new way to improve the positioning accuracy of pedestrian navigation in an indoor GNSS-denied environment.

## Figures and Tables

**Table 1:** The effect of gyro-accelerometer asynchronous time on pedestrian navigation under different motions.

-	Pitch	Roll	Yaw
$\delta v_{E}^{n}$	$\tau \sin\psi_{0}\int_{0}^{t}\omega_{\theta }f_{U}^{n}dt$	$\tau \cos\psi_{0}\int_{0}^{t}\omega_{\gamma }f_{U}^{n}dt$	$-\tau \int_{0}^{t}\omega_{\psi }f_{N}^{n}dt$
$\delta v_{N}^{n}$	$-\tau \cos\psi_{0}\int_{0}^{t}\omega_{\theta }f_{U}^{n}dt$	$\tau \sin\psi_{0}\int_{0}^{t}\omega_{\gamma }f_{U}^{n}dt$	$\tau \int_{0}^{t}\omega_{\psi }f_{E}^{n}dt$
$\delta v_{h}^{n}$	$\tau \int_{0}^{t}\omega_{\theta }f_{U}^{n}dt$	$\tau \int_{0}^{t}\omega_{\gamma }f_{U}^{n}dt$	$\tau \sqrt{{\left(\int_{0}^{t}\omega_{\psi }f_{N}^{n}dt\right)}^{2}+{\left(\int_{0}^{t}\omega_{\psi }f_{E}^{n}dt\right)}^{2}}$
$\delta v_{U}^{n}$	$\tau \int_{0}^{t}\omega_{\theta }f_{//}^{n}dt$	$-\tau \int_{0}^{t}\omega_{\gamma }f_{⊥}^{n}dt$	0
δL	$-\frac{\tau \cos\psi_{0}}{R_{M}+h}\int_{0}^{t}\int_{0}^{t}\omega_{\theta }f_{U}^{n}dtdt$	$\frac{\tau \sin\psi_{0}}{R_{M}+h}\int_{0}^{t}\int_{0}^{t}\omega_{\gamma }f_{U}^{n}dtdt$	$\frac{\tau }{R_{M}+h}\int_{0}^{t}\int_{0}^{t}\omega_{\psi }f_{E}^{n}dtdt$
δλ	$\frac{\tau \sin\psi_{0}\sec L}{R_{N}+h}\int_{0}^{t}\int_{0}^{t}\omega_{\theta }f_{U}^{n}dtdt$	$\frac{\tau \cos\psi_{0}\sec L}{R_{N}+h}\int_{0}^{t}\int_{0}^{t}\omega_{\gamma }f_{U}^{n}dtdt$	$\frac{-\tau \sec L}{R_{N}+h}\int_{0}^{t}\int_{0}^{t}\omega_{\psi }f_{N}^{n}dtdt$
δh	$\tau \int_{0}^{t}\int_{0}^{t}\omega_{\theta }f_{//}^{n}dtdt$	$-\tau \int_{0}^{t}\int_{0}^{t}\omega_{\gamma }f_{⊥}^{n}dtdt$	0

**Table 2:** The main characteristics of velocity errors under different motions.

Motion	Horizontal Velocity Error	Vertical Velocity Error
Pitch motion	The errors will cancel each other out in opposite directions	Increase
Roll motion	The errors will cancel each other out in opposite directions	Increase
Yaw motion	The errors will cancel each other out in opposite directions	Make no difference
Walking on flat ground	The errors will cancel each other out when walking on a closed-loop trajectory	Increase
Going upstairs	The errors will cancel each other out when walking on a closed-loop trajectory	Increase
Going downstairs	The errors will cancel each other out when walking on a closed-loop trajectory	Increase

**Table 3:** Performance of MIMU.

Performance	Gyros	Accelerometers
In-run stability	$10^{\circ }$/h	40 ug
Random walk	$0.4^{\circ }$/$\sqrt{}h$	0.06 m/s /$\sqrt{}h$
Full range	$\pm 2000^{\circ }$/h	±40 g

**Table 4:** Experimental methods.

Method	Detail
Method 1	ZUPT with gyro-accelerometer asynchronous time ignored
Method 2	ZUPT with gyro-accelerometer asynchronous time considered
Method 3	ZUPT/height constraint with gyro-accelerometer asynchronous time ignored
Method 4	ZUPT/height constraint with gyro-accelerometer asynchronous time considered

**Table 5:** Comparison of position errors by different methods ($P_{1}$).

Errors	Method 1	Method 2	Method 3	Method 4
North position error (m)	0.38	0.35	0.17	0.18
East position error (m)	0.36	0.23	0.27	0.15
Horizontal position error (m)	0.52	0.42	0.32	0.23
Error percentage (%D)	0.50	0.40	0.31	0.22
Height error (m)	1.03	0.76	0.08	0.06

**Table 6:** Comparison of position errors by different methods ($P_{2}$).

Errors	Method 1	Method 2	Method 3	Method 4
North position error (m)	0.93	0.75	0.44	0.36
East position error (m)	1.06	0.92	1.01	0.85
Horizontal position error (m)	1.41	1.19	1.10	0.92
Error percentage (%D)	0.70	0.59	0.54	0.46
Height error (m)	2.48	1.36	0.20	0.18

**Table 7:** Comparison of position errors by different methods ($P_{3}$).

Errors	Method 1	Method 2	Method 3	Method 4
North position error (m)	1.70	1.38	0.89	0.66
East position error (m)	1.84	1.60	1.66	1.43
Horizontal position error (m)	2.51	2.11	1.88	1.58
Error percentage (%D)	0.84	0.70	0.64	0.53
Height error (m)	3.37	2.11	0.22	0.23
